# Peruvian healthcare system readiness for dementia: First insights from an ongoing research study

**DOI:** 10.1002/alz.088535

**Published:** 2025-01-09

**Authors:** Francisco Tateishi Serruto, Maria Lazo Porras, Jemma Hawkins, María Sofía Cuba, Christopher Butler, Antonio Bernabe, Graham Moore, Filipa Landeiro, Maria Kathia Cardenas, Francisco Diez Canseco, Miriam Lucar Flores, Silvana Perez León Quinoso

**Affiliations:** ^1^ Cronicas, Universidad Peruana Cayetano Heredia, LIma, Lima Peru; ^2^ CRONICAS, Universidad Peruana Cayetano Heredia, Lima Peru; ^3^ Cardiff University, Cardiff United Kingdom; ^4^ Universidad Peruana Cayetano Heredia, Lima, Lima Peru; ^5^ Department of Brain Sciences, Imperial College London, London United Kingdom; ^6^ Cardiff University, Cardiff, Wales United Kingdom; ^7^ University of Oxford, Oxford United Kingdom; ^8^ CRONICAS, Universidad Peruana Cayetano Heredia, Lima, Lima Peru; ^9^ CRONICAS, Universidad Peruana Cayetano Heredia, Perú, Lima Peru

## Abstract

**Background:**

Peru does not have official prevalence data of dementia, however, particular studies indicate that in urban areas 6.85% of the population over 65 years of age has it. Countries such as Peru have significant drivers of the condition such as low socio‐economic (monetary poverty 27.5%) and educational levels (21.9% of the population has only primary education). In order to prepare the health system and society in general, it is necessary to start multisectoral studies to understand the complexity of the challenge ahead. The IMPACT project aims to contribute to this.

**Method:**

Mixed methods approach. Semi‐structured interviews with different stakeholders as well as secondary data review to cover 11 themes from the health system (policy environment, financing, infrastructure, service delivery in prevention and management issues, etc.) in three levels (macro, meso and micro) nnd in four different areas of Peru

**Result:**

The presentation still does not show results, as it is still ongoing. However, we are sharing some of the insights and learnings we have gained so far. Some of these are the following: limited and heterogeneous response due to the highly fragmented system, lack of political support, first level of care not designed or prepared to deal with dementia cases, reduced amount of specialists, adequate training for health providers attending this group, no guidelines for medical practices and limited research about its characteristics and needs. Also, age stereotypes and lack of awareness about dementia as a medical condition persist in all levels of the health system and society, negatively affecting the effectiveness of its response to their needs

**Conclusion:**

These are some preliminary Conclusions: more research is needed, to address stereotypes, increase training for health providers, use technology to facilitate access to services, create models of effective implementation to generate impact on urgent issues and inclusion of preventive and long‐term care approaches

